# Lymphatic Imaging and Interventions in Children: Current Practice

**DOI:** 10.1007/s00270-026-04582-5

**Published:** 2026-08-24

**Authors:** C. C. Pieper, D. Rabinowitz, G. Maleux, A. Srinivasan

**Affiliations:** 1 Division for Minimally Invasive Lymphatic Therapy, Department of Diagnostic and Interventional Radiology, University Hospital Bonn, Venusberg-Campus 1, 53127 Bonn, Germany; 2 Department of Radiology, Division of Interventional Radiology, Nemours Children’s Health, Wilmington, DE USA; 3 Universitair Ziekenhuis Leuven, Herestraat 49, 3000 Leuven, Belgium; 4 Department of Radiology, Children’s Hospital of Philadelphia, Philadelphia, PA USA

**Keywords:** Lymphatic imaging, Lymphatic interventions, Pediatrics, Magnetic resonance lymphangiography, Lymphangiography, Congenital heart disease

## Abstract

**Purpose:**

To provide a decision-oriented overview of clinical manifestations, imaging strategies, and minimallyinvasive treatment of pediatric lymphatic flow disorders.

**Materials and Methods:**

Current practice in fluoroscopic lymphangiography, dynamic contrast-enhanced magneticresonance lymphangiography, and image-guided interventions was reviewed, emphasizing congenital heart disease,complex lymphatic anomalies, procedural adaptations, and safety.

**Results:**

Lymphatic flow disorders can cause chylous eff usions, plastic bronchitis, protein-losing enteropathy, andlymphedema. Modern imaging enables high-resolution assessment of anatomy and abnormal flow and guidesselective interventions. Small clinical series report favorable outcomes after selective embolization of abnormallymphatic channels; however, inappropriate or nonselective embolization may worsen lymphatic failure.

**Conclusions:**

Accurate clinical characterization, tailored imaging, and treatment in experienced multidisciplinarycenters are essential for safe, individualized management.

## Introduction

**Fig. 1:**
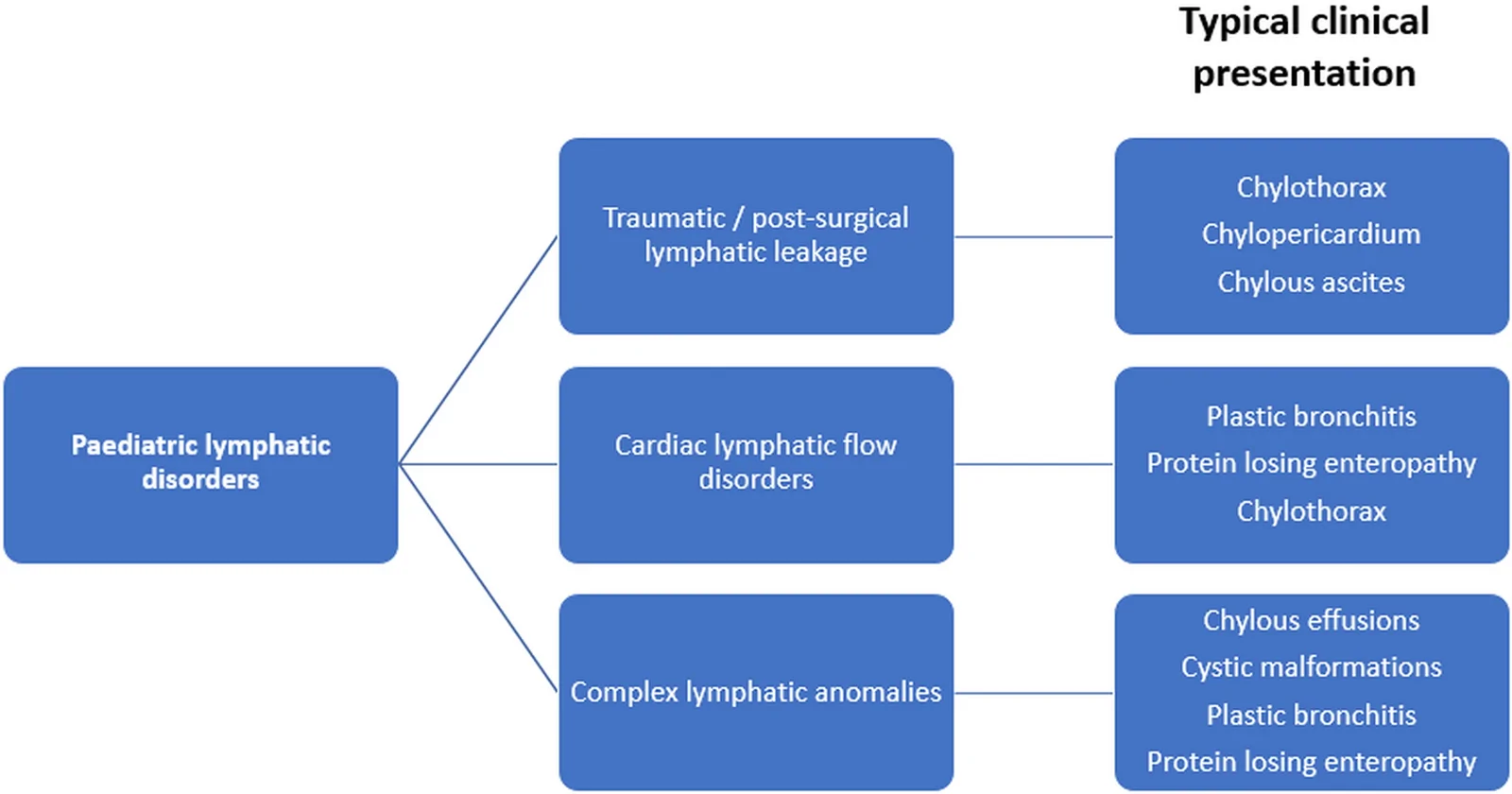
Pediatric lymphatic disorders overview

Pediatric lymphatic disorders comprise a diverse spectrum of rare but clinically severe conditions associated with lymphatic leakage or impaired lymphatic transport and require a high index of suspicion for diagnosis [1, 2]. Lymphatic leaks may be traumatic/iatrogenic or may result from lymphatic flow disorders. The latter are caused by obstruction of lymphatic flow, lymphatic dysplasia, or both and can be categorized as (i) secondary, most commonly after surgical palliation of single-ventricle congenital heart disease (CHD) and less commonly in infants with severe chronic lung disease of prematurity, or (ii) primary, including complex lymphatic anomalies. Whereas post-traumatic leaks are more common in adults, lymphatic flow disorders associated with CHD and complex lymphatic anomalies represent an important proportion of pediatric lymphatic disease. Key clinical presentations and associations relevant to children and adolescents are summarized in Fig. 1.

Understanding and early clinical recognition of these conditions are essential for appropriate imaging work-up and multidisciplinary treatment. For the interventional radiologist, familiarity with the clinical presentations helps guide diagnostic strategy and procedural planning. Because of their complexity, patients with suspected lymphatic flow disorders should be referred early to a center with multidisciplinary experience in lymphatic imaging, lymphatic interventions, pediatric anesthesia, and medical and surgical management. In contrast to broader reviews of central lymphatic imaging, this article provides a pediatric, decision-oriented framework that links clinical presentation and pathophysiology to the appropriate lymphatic compartment, technical lymphatic imaging approach, and selective interventional strategy. Particular emphasis is placed on pediatric procedural adaptations and safety considerations in CHD and other conditions with potential right-to-left shunting.

## Clinical Presentations and Pathophysiologic Patterns

### Chylous Effusions

Chylous fluid (i.e., lymph containing chylomicrons and triglycerides > 110 mg/dL on normal diet) can accumulate in different compartments, including the pleural, peritoneal, and pericardial spaces. Chylothorax, the accumulation of lymphatic fluid in the pleural cavity, is the most common lymphatic complication in children, especially in those with single-ventricle physiology. It may also occur spontaneously in complex lymphatic anomalies. The presence of a chylous effusion does not necessarily indicate a rapid focal leak, because lymph may also seep diffusely, for example through pleural stomata in pulmonary lymphangiectasia. The suspected mechanism, rather than the chylous appearance alone, should therefore guide subsequent imaging and treatment planning [1–3]. Detailed management strategies are discussed below.

### Plastic Bronchitis

**Fig. 2:**
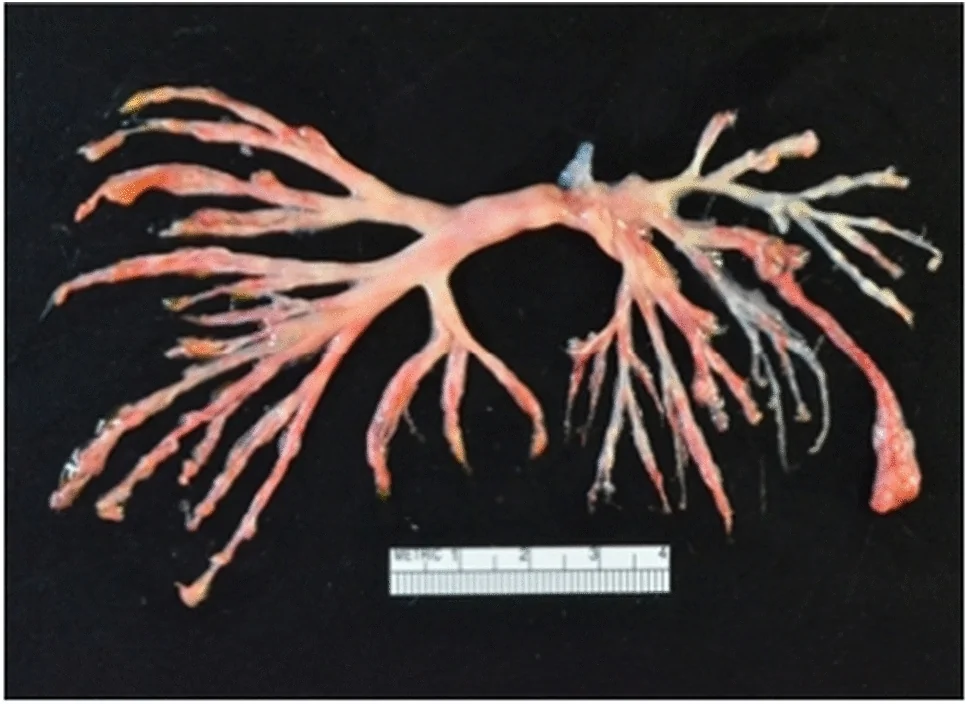
Pathologic specimen of a large proteinaceous bronchial cast obtained from post-mortem examination of a patient with plastic bronchitis

Plastic bronchitis (PB) results from abnormal pulmonary lymphatic perfusion, with protein-rich lymph entering the bronchial submucosa and airway lumen, where inspissated material forms casts that may cause airway plugging, chronic cough, cast expectoration, and potentially life-threatening obstruction (Fig. 2). In children, PB is most frequently associated with Fontan circulation, in which elevated central venous pressure impedes thoracic duct (TD) drainage and promotes decompressive lymphatic channels toward the airways. Dynamic lymphatic imaging can demonstrate this abnormal flow pattern and identify the culprit peribronchial lymphatic channels [2, 4]. Cross-sectional CT may demonstrate airway casts or secondary atelectasis, but it does not characterize the causative lymphatic flow pattern.

### Protein-Losing Enteropathy

Protein-losing enteropathy (PLE) results from lymphatic leakage into the gastrointestinal tract and is most frequently observed after Fontan palliation. Increased lymphatic afterload causes decompressive duodenal or jejunal lymphatics to become dilated and incompetent, allowing protein-rich hepatic lymph to enter the bowel lumen. This results in chronic diarrhea, abdominal pain, wasting, and systemic protein depletion. PLE is supported by an increased stool alpha-1-antitrypsin concentration and a low serum albumin concentration (< 3 g/dL). Imaging must determine whether hepatic, mesenteric, or combined lymphatic pathways contribute to the leak [2, 4–6].

### Lymphedema and Anasarca

Lymphedema is characterized by accumulation of excess interstitial fluid, particularly within subcutaneous tissues, resulting in swelling of an extremity or another body region. Anasarca refers to generalized or predominantly truncal subcutaneous edema. Lymphedema should be differentiated clinically from fatty overgrowth and other causes of edema before dedicated lymphatic imaging is performed [7]. Cardiogenic edema, which may occur in patients with Fontan circulation or lymphatic failure, often presents as anasarca. In children, lymphedema is more often associated with complex lymphatic anomalies than with single-ventricle CHD. Unlike lymphatic leaks in the chest or abdomen, there is no established percutaneous intervention for isolated peripheral lymphedema; management focuses on compression, physiotherapy, targeted medical therapy when appropriate, and, in selected cases, lymphovenous anastomosis or vascularized lymph node transfer.

## Lymphatic Imaging Modalities

The field of lymphatic imaging and intervention has expanded rapidly over the past decade, particularly in pediatric patients, in whom lymphatic flow disorders are important causes of morbidity. For interventional radiologists unfamiliar with this field, the lymphatic system may appear enigmatic because of its anatomical variability, absence of an actively pumped closed-loop circulation, and historically limited imaging capabilities. Emerging diagnostic tools now permit direct assessment of lymphatic anatomy and flow and have become essential for selecting the appropriate access and planning lymphatic interventions.

### Fluoroscopic Lymphangiography

Fluoroscopic lymphangiography has been the reference standard for lymphatic imaging for decades and provides high-spatial-resolution visualization of lymphatic vessels and abnormalities [1]. It can be performed through pedal, nodal, mesenteric, or hepatic access. Access is obtained with a 22- to 25-gauge (G) needle under ultrasound (US) guidance. Needle position can be confirmed fluoroscopically by injection of water-soluble contrast material. Once access is confirmed, ethiodized oil (Lipiodol, Guerbet, France; or Vividol, Vivere Imaging, India) is injected at a recommended maximum dose of 0.25 mL/kg and a rate of approximately 0.2 mL/min. The principal advantage of oily contrast material is its persistent visibility within lymphatic fluid. Important limitations include the risk of systemic arterial, including cerebral, oil embolization in patients with right-to-left shunts, such as a Fontan fenestration or veno-venous collaterals. Non-water-soluble agents should therefore be avoided unless relevant shunts have been excluded or temporarily protected. Fluoroscopic lymphangiography remains indispensable for interventions but has lower contrast resolution and provides a less comprehensive field-of-view assessment than dynamic contrast-enhanced MR lymphangiography (DCMRL) [2].

### Dynamic Contrast-Enhanced Magnetic Resonance Lymphangiography

DCMRL is performed by off-label intralymphatic injection of a gadolinium-based contrast medium at one or more access sites. The examination usually begins with heavily T2-weighted imaging to depict fluid distribution and static lymphatic fluid in dilated channels (non-contrast MRL). This sequence is best suited for evaluating TD morphology, lymphatic malformations, and chylous effusions but does not provide directional flow information. Heavily T2-weighted imaging alone may be used for selected preoperative assessments or when contrast administration is not feasible or has been unsuccessful [8]. Breath-holding is usually impractical in infants and young children; respiratory-triggered or navigator-gated acquisitions, with cardiac gating when appropriate, are therefore preferred to reduce motion artifacts.

Subsequent contrast-enhanced sequences require prior US- and/or fluoroscopy-guided access to inguinal lymph nodes, hepatic lymphatics, and/or mesenteric lymphatics, which has a high technical success rate in adults and children [9, 10]. DCMRL provides time-resolved, near-real-time, radiation-free images with high contrast resolution and is instrumental in characterizing lymphatic anatomy and flow dynamics. Access at multiple sites can be helpful for characterizing lymphatic flow disorders, as it permits multi-compartment assessment through intranodal, intrahepatic, and intramesenteric contrast injection [11, 12]. General anesthesia is often required in infants and young children, whereas older cooperative children may be examined under sedation or without anesthesia, depending on clinical status and institutional workflow. Access is obtained with appropriately sized 22- to 25-G needles. Inguinal lymph nodes are accessed bilaterally under US guidance; needle position can be confirmed by fluoroscopic injection of water-soluble contrast material, particularly in a hybrid angiography-MR suite, or by injection of an US contrast agent or saline under US guidance. For hepatic lymphatic access, a long 25-G needle is advanced under US guidance into the echogenic tissue anterior to the main portal vein, and access is verified as described above. Hepatic access is particularly informative in PLE and in PB or chylothorax when hepatopulmonary lymphatic connections are suspected. Mesenteric access, which is especially useful for PLE and chylous ascites, is obtained by puncturing mesenteric lymphatics adjacent to the central superior mesenteric vein or a mesenteric lymph node. Because bowel may be traversed, prophylactic antibiotics, commonly ceftriaxone and metronidazole, are administered. The needles are secured before the patient is carefully transported to the MRI suite.

Following heavily T2-weighted imaging, time-resolved MR angiography can be performed sequentially at each access site during slow injection of diluted gadolinium-based contrast medium. The total gadolinium dose for the complete examination, including all access sites, is typically up to 0.2 mmol/kg and is diluted 1:5 to 1:10 in saline. A representative protocol includes approximately 25 acquisitions per access site, each lasting 8–12 s. Intralymphatic administration of gadolinium-based contrast medium is off-label. In patients with renal impairment, ferumoxytol at a dose of 3 mg/kg in a similar dilution may be considered as an off-label alternative, with attention to the small risk of anaphylaxis and subsequent hypersensitivity [13].

After time-resolved MR lymphangiography, a static fat-suppressed 3D T1-weighted gradient-echo sequence is obtained and provides higher contrast resolution than the time-resolved acquisition. DCMRL is particularly suited for detecting abnormal lymphatic pathways, obstruction, reflux, and leakage. Although highly informative, it requires close workflow coordination among interventional radiology, MRI, and anesthesia teams.

### Computed Tomographic Lymphangiography

CT lymphangiography (CTL) is an emerging modality performed after intranodal injection of water-soluble iodinated contrast medium. It is logistically less complex than DCMRL [14, 15] and provides high spatial resolution with 3D reconstructions. Its use in children should be restricted because of ionizing radiation and the absence of truly dynamic flow information; repeated CT acquisitions for dynamic assessment would result in an unacceptable radiation burden. CTL may nevertheless be useful when MRI is unavailable or contraindicated, when metallic implants cause artifacts that preclude adequate DCMRL interpretation, or when precise high-spatial-resolution delineation of TD anatomy is required before intervention. Pediatric protocols should be strictly dose optimized, and DCMRL remains preferred when feasible, particularly in small children.

### Lymphoscintigraphy

Lymphoscintigraphy involves intradermal injection of technetium-99 m-labeled sulfur colloid, nanocolloid, or tilmanocept (Cardinal Health, USA), followed by serial planar or SPECT imaging to track lymphatic drainage [16]. Even when fused with CT images, it has limited spatial and contrast resolution for visualization of the central lymphatics and is rarely used for procedural planning. Future development of dedicated tracers may improve evaluation, particularly of mesenteric lymphatics.

### Near-Infrared Fluorescence Imaging

Near-infrared fluorescence imaging involves intradermal injection of indocyanine green and depicts superficial lymphatics to a depth of approximately 1.5 cm. It may be valuable for planning lymphatic reconstructive surgery for lymphedema but is not suitable for assessing the central lymphatic system.

No single modality answers all clinical questions. DCMRL is preferred for comprehensive time-resolved assessment of central lymphatic anatomy and flow; fluoroscopic lymphangiography remains indispensable when intervention is anticipated; CTL is reserved for selected anatomical or technical scenarios; and lymphoscintigraphy and near-infrared fluorescence imaging primarily address peripheral rather than central lymphatics. A practical selection framework is summarized in Table 1.

**Table 1:** Practical selection of lymphatic imaging modalities in children

Clinical question or scenario	Preferred imaging approach	Key decision points and alternatives
Central anatomy, TD morphology, suspected complex lymphatic anomaly	Heavily T2-weighted non-contrast MRL followed by DCMRL when flow information is required	Use multi-compartment access if the suspected source is not captured by intranodal imaging; non-contrast MRL alone does not show flow direction
Chylothorax or plastic bronchitis with suspected pulmonary lymphatic perfusion	Intranodal DCMRL for mapping; fluoroscopic lymphangiography when intervention is planned	Add intrahepatic imaging if no TD-derived pathway is identified or a hepatopulmonary connection is suspected. Assess right-to-left shunts before oil or glue administration
Protein-losing enteropathy	Hepatic fluoroscopic lymphangiography / hepatic DCMRL	Add intramesenteric DCMRL or mesenteric lymphangiography when frank chyle leakage suggests a mesenteric contribution or hepatic imaging is incomplete
Persistent postoperative chyle leak	Heavily T2-weighted non-contrast MRL for lymphatic anatomy; DCMRL to distinguish focal leakage from impaired run-off, followed by fluoroscopic lymphangiography for treatment	CTL or cone-beam CT may supplement target localization when MRI is unavailable, degraded by metal, or insufficiently precise for direct puncture
Peripheral lymphedema or planning of reconstructive lymphatic surgery	Peripheral MRL; lymphoscintigraphy and/or near-infrared fluorescence imaging	These techniques primarily assess peripheral drainage and/or superficial collectors; central DCMRL is reserved for suspected systemic or central lymphatic disease
MRI contraindication, severe MR artifact, or need for high-spatial-resolution TD anatomy	Dose-optimized CTL	Reserve for selected pediatric cases because it lacks comprehensive dynamic flow information and uses ionizing radiation

CTL, CT-lymphangiography; DCMRL, dynamic contrast-enhanced MR-lymphangiography; TD, thoracic duct

## Management of Lymphatic Disorders

Initial management is presentation specific and may include drainage of symptomatic effusions, nutritional modification, diuretics, steroids or somatostatin analogs in selected patients, and physiotherapy. Additional inhaled therapies are used for PB, whereas compression and reconstructive surgery apply to peripheral lymphedema. The following sections focus on when advanced imaging and image-guided or hybrid treatment should be considered.

### Postoperative Lymphatic Leak

**Fig. 3:**
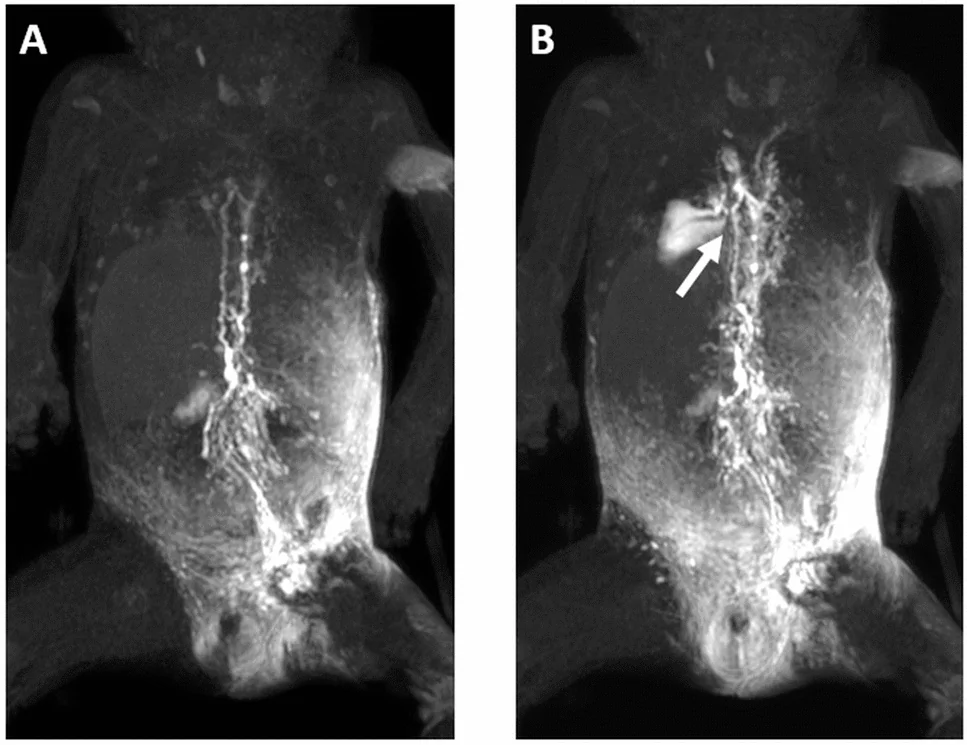
Postoperative lymphatic leakage. Maximum-intensity projections (MIPs) of DCMRL at an early (**A**) and later phase (**B**) in a 4-week-old preterm boy with right-sided postoperative chylothorax after repair of esophageal atresia. DCMRL demonstrates abnormal central lymphatic anatomy with impaired lymphatic run-off, extensive reflux into dermal lymphatics, and active leakage into the right pleural cavity (arrow)

Postoperative chylothorax and chylous ascites are uncommon in children; chylothorax occurs in an estimated 1–4% of patients after pediatric cardiothoracic surgery [17]. Postoperative chyle leaks may cause serious sequelae, including malnutrition, electrolyte imbalance, immunodeficiency, and respiratory compromise. Initial treatment consists of conservative measures, including percutaneous drainage, dietary modification, and administration of somatostatin analogs [17, 18]. Diagnostic work-up includes imaging of postoperative collections and analysis of the fluid. A milky-white appearance and triglyceride levels > 110 mg/dL strongly suggest chylous content. Postoperative chylothorax in children has been described after congenital heart surgery [17–19], thoracic spine surgery [17], and surgery for esophageal atresia or tracheoesophageal fistula (Fig. 3) [17, 20].

Postoperative chylous ascites is less frequent in children and has been described after liver transplantation [21], retroperitoneal resection of neuroblastoma [19, 20], and surgery for lymphatic malformations [19].

If postoperative chylous effusions persist despite optimized conservative measures, particularly with high-volume output (> 20 mL/kg/day) or persistence for more than 10 days, interventional and surgical consultation is justified. DCMRL can delineate the lymphatic anatomy and distinguish a focal leak from impaired central run-off before intervention. Depending on the mechanism, intranodal ethiodized-oil lymphangiography may be used for its potential therapeutic effect, particularly in persistent lower-output leaks [22], or to guide direct transabdominal puncture of the leaking lymphatic vessel, such as the TD, followed by embolization with a liquid embolic agent. If imaging demonstrates no focal target but suggests TD obstruction, relief of the obstruction should be considered before embolization to avoid exacerbating lymphatic failure.

In a case series [17] of 11 pediatric patients with refractory postoperative chylothorax, central lymphatics were visualized in 8 of 12 procedures after ethiodized oil was injected into inguinal lymph nodes. The TD was cannulated and embolized in 5 of these 8 procedures, and TD disruption by multiple punctures was performed in the remaining 3. Overall clinical success was achieved in 64% of patients without major adverse events. Gupta et al. successfully treated one patient with chylothorax after a Glenn procedure and two patients with postoperative chylous ascites using bilateral inguinal intranodal injection of ethiodized oil [19]. Verhaeghe et al. similarly managed refractory post-transplant chylous ascites in a 9-year-old girl [21]. Woerner et al. treated retroperitoneal chyle leaks after neuroblastoma resection in two children by fluoroscopic lymphangiography and cone-beam CT-guided direct puncture, followed by embolization with n-butyl cyanoacrylate (nBCA; typical dilution, 25% in ethiodized oil) [23].

Hybrid interventional and surgical procedures are an alternative for refractory postoperative chylothorax when percutaneous treatment is unsuccessful or central lymphatic vessels are too small to cannulate. Kumar et al. described fluoroscopic lymphangiography followed by pleurodesis with 4% povidone-iodine through a chest tube [18], and Shirotsuki et al. used indocyanine-green fluorescence lymphangiography to guide thoracoscopic TD ligation [20].

### Lymphatic Failure in Patients with Congenital Heart Disease

Patients with CHD, most commonly those with single-ventricle physiology after Fontan palliation, constitute a large population of children with lymphatic flow disorders. PB, chylothorax, and PLE are the major manifestations [24], and lymphatic failure occurs in approximately 4–10% of cases. Elevated central venous pressure increases both lymphatic afterload, defined as resistance to lymphatic egress from the TD into the central veins, and lymph production. Experimental data demonstrate an exponential increase in lymphatic flow as pressure rises at the TD-venous junction [25]. This combination promotes lymphatic seepage or leakage through aberrant channels into the bronchi, pleural space, or bowel [24]. Interventional lymphatic approaches have been developed for these severe complications, which previously could be treated only conservatively or by cardiac transplantation [26].

Before lymphatic imaging or intervention in a patient with Fontan circulation, the relevant cardiac and venous anatomy must be reviewed. Many Fontan circuits have a fenestration connecting the Fontan tunnel to the systemic atrium. Veno-venous collaterals from the central veins to the pulmonary veins are also common and create additional right-to-left shunts. Consequently, non-water-soluble agents introduced into the lymphatic system may reach the arterial circulation and cause end-organ ischemia [27]. Risk-reduction strategies include preprocedural embolization of veno-venous collaterals and temporary balloon occlusion of a Fontan fenestration during administration of oil-based contrast material or glue. Metallic implants, including MR-incompatible pacemakers, may contraindicate MRI or substantially degrade DCMRL. Comorbid circulatory failure and thrombophilia must also be considered during procedural planning and recovery.

### Plastic Bronchitis and Chylothorax

**Fig. 4:**
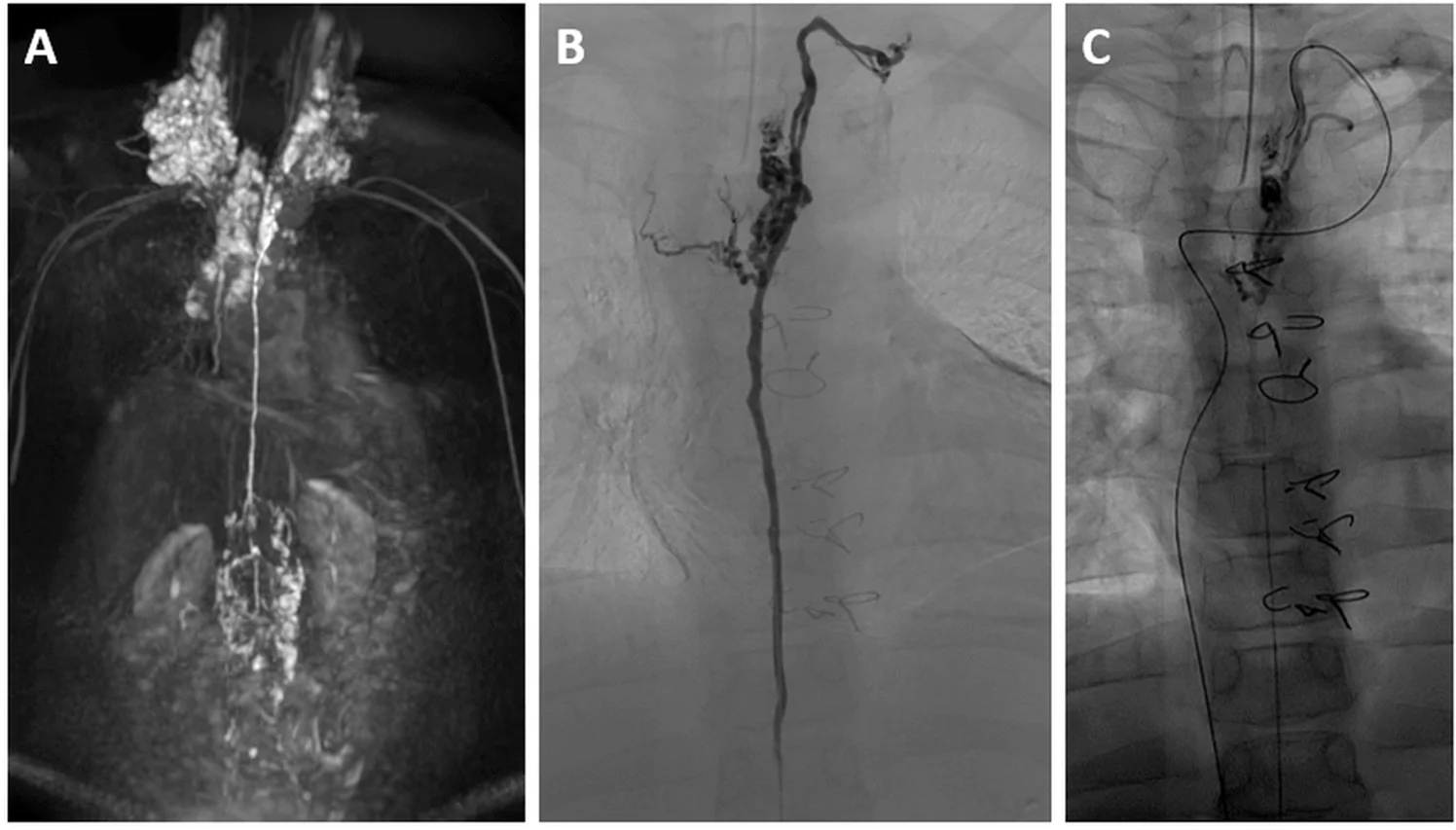
5-year-old male with Fontan and plastic bronchitis. (**A**) MIP of DCMRL demonstrating a dilated thoracic duct and mediastinal/peribronchial lymphatic perfusion. (**B**) Fluoroscopic lymphangiogram with a 2.4Fr microcatheter in the thoracic duct with contrast injection demonstrating aberrant flow to the right paratracheal and right peribronchial region. (**C**) Subselective glue embolization via microcatheter advanced from the venous side secondary to the angle of vessel takeoff from the thoracic duct. Control microcatheter is in the thoracic duct

The reported incidence of PB in patients with Fontan circulation is 5–10%, and mortality has been reported to be as high as 50% [28]. Pulmonary lymphatic perfusion can be demonstrated by DCMRL and fluoroscopic lymphangiography; abnormal channels typically arise from the TD and perfuse mediastinal and peribronchial lymphatics (Fig. 4A) [29]. DCMRL is used for procedural planning and is followed, when intervention is indicated, by percutaneous TD lymphangiography and access. Supportive treatment may include a low-fat diet, diuretics, steroids or somatostatin analogs in selected patients, physiotherapy, and drainage of symptomatic effusions. For PB, inhaled mucolytics, including N-acetylcysteine or tissue plasminogen activator, and bronchodilators may be added.

The TD is first opacified with water-soluble contrast material injected into hepatic, mesenteric, or retroperitoneal lymphatics through a 25-G needle. A second 22- or 25-G needle of appropriate length, advanced at an angle of 45 degrees or less, is guided into the TD near the cisterna chyli at the T12-L1 vertebral level. A 0.010-inch wire for a 25-G needle or a 0.014-inch wire for a 22-G needle is advanced into the central TD and is serially upsized to 0.018 inch through catheter exchange; a 2.4-F microcatheter is then used for intervention. The target lymphatic network responsible for pulmonary lymphatic perfusion is cannulated with an angled 0.014- or 0.018-inch wire. Selective embolization is typically performed with nBCA diluted in iodized oil. Coils may be used as an adjunct, particularly in the uncommon setting of complete TD embolization. Blue dye, such as Patent Blue V (Guerbet, France), can be injected into the TD during bronchoscopy (Fig. 4B). If blue dye is identified in the airway, the leaking collaterals are selectively embolized with a liquid embolic agent (Fig. 4C). Embolization must be performed cautiously to avoid nontarget passage into veno-venous collaterals or reflux into the TD. Complete TD embolization should be considered only when selective embolization is technically infeasible because further impairment of central lymphatic outflow may cause severe complications such as PLE or ascites [30]. A clinical success rate of 94% has been reported for lymphatic embolization in PB in a selected cohort [3], and successful emergency treatment has also been described [31]. A major procedural risk is nontarget glue embolization into venous channels that communicate with the arterial circulation.

Occasionally, no TD-derived pulmonary lymphatic perfusion is identified. In these cases, hepatic lymphangiography may demonstrate abnormal hepatopulmonary lymphatic connections to the airways [32]. Retrograde TD access from the subclavian vein has also been reported with successful outcomes [33], but variant TD outlets are common in this population and should be evaluated on DCMRL before the procedure.

### Protein-Losing Enteropathy

**Fig. 5:**
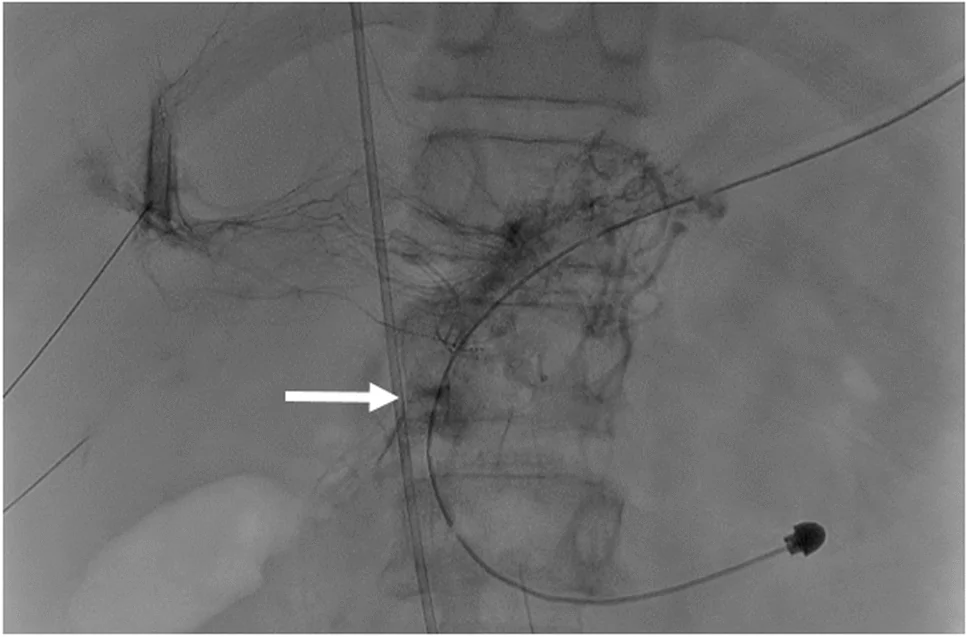
A 16-year-old boy with a fenestrated Fontan circulation and severe PLE. Water-soluble contrast injection via 25G needle in the liver lymphatics demonstrates aberrant lymphatic vessels leaking directly into the lumen of the duodenum (arrow), localized by the nasoduodenal tube

PLE presents with edema, immunodeficiency, thrombophilia, and diarrhea [34], and an incidence of up to 24% has been reported after Fontan palliation [35]. Conservative treatment includes a low-fat diet, diuretics, albumin replacement, corticosteroids, and midodrine [34, 36]. The principal mechanism in Fontan-associated PLE is leakage of hepatic lymph into the duodenum through abnormal lymphatic vessels, which can be depicted by hepatic lymphangiography [35]. As for hepatic DCMRL access, a 25-G needle is advanced under US and fluoroscopic guidance into the periportal lymphatics anterior to the main portal vein, and water-soluble contrast material is injected. Abnormal lymphatic vessels may be seen approaching the duodenum, sometimes with direct contrast leakage into the bowel lumen (Fig. 5). Leakage can be confirmed by blue-dye injection during concomitant endoscopy. The abnormal channels can then be embolized with nBCA through an antegrade hepatic or periduodenal approach or through a retrograde transendoscopic approach [35, 37].

Occasional frank chyle leakage into the duodenum indicates a contribution from mesenteric lymphatics; intramesenteric DCMRL or fluoroscopic mesenteric lymphangiography is therefore required for complete assessment. Injection of large volumes of ethiodized oil near or into the duodenum should be minimized because gastrointestinal bleeding has been reported [35]. Recurrent PLE after initially successful embolization is common [6, 35, 37]. Repeat embolization is technically feasible, although evidence regarding durable long-term efficacy remains limited.

### Complex Lymphatic Anomalies

Complex lymphatic anomalies are rare but often debilitating developmental disorders of the lymphatic system. According to the International Society for the Study of Vascular Anomalies classification, they include generalized lymphatic anomaly (GLA), kaposiform lymphangiomatosis (KLA), Gorham-Stout disease (GSD), and central conducting lymphatic anomaly (CCLA). Reliable population-based incidence estimates for the individual complex lymphatic anomalies are not available; published evidence consists largely of small registries and case series [38, 39]. Advances in lymphatic imaging, gene profiling, and minimally invasive lymphatic interventions have broadened the diagnostic and therapeutic options for these conditions and permit improved disease characterization and increasingly individualized treatment.

**Fig. 6:**
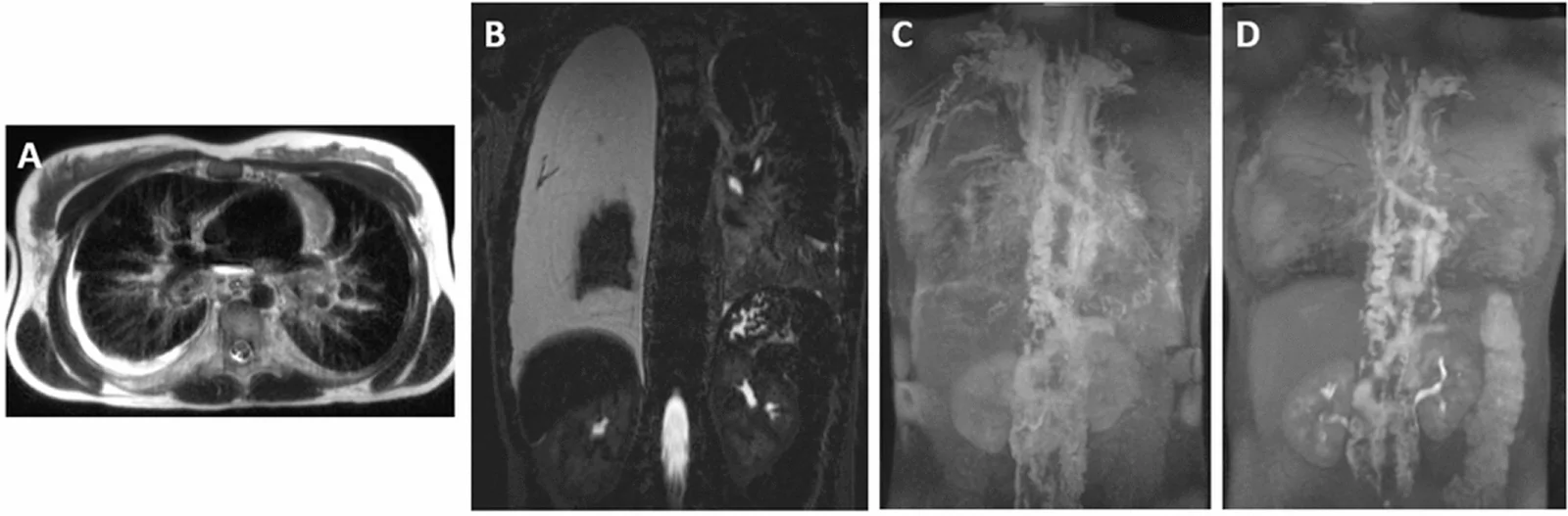
18-year-old female with kaposiform lymphangiomatosis due to CBL mutation. (**A**) Axial T2-weighted MRI showing infiltrating lymphatic malformation in the mediastinum, hila, and pulmonary interstitium (arrows). (**B**) Coronal T2-weighted MRI showing large right pleural effusions and interstitial infiltration of lymphatic malformation on the left. (**C**) MIP of coronal T1-weighted gradient echo sequences after injection of contrast into inguinal nodes showing a markedly disorganized lymphatic system and severe bilateral pulmonary lymphatic perfusion that significantly remodeled after 15 months on MEK inhibitor therapy (**D**)

**Fig. 7:**
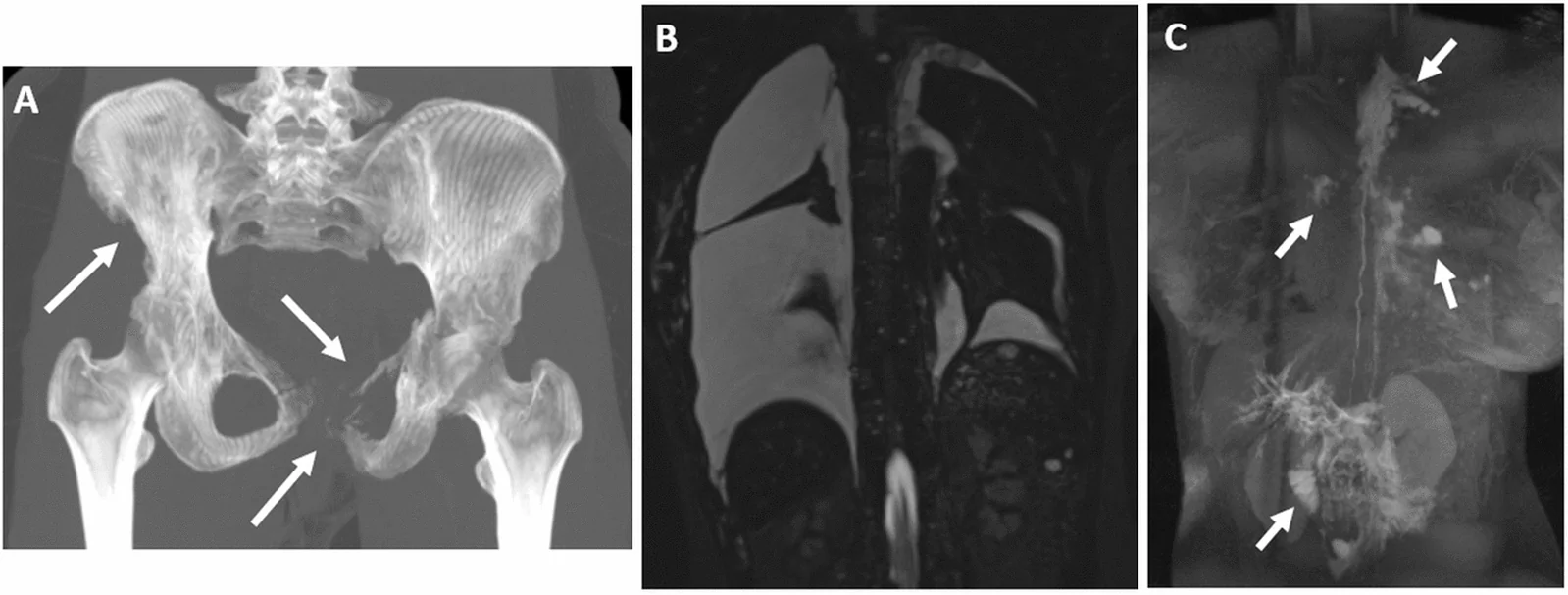
23-year-old female with Gorham-Stout disease, protein-losing enteropathy, and large chylous effusions. (**A**) Coronal MIP of CT scan showing osteolysis of the pubic bones and the right ilium (long arrows). (**B**) Coronal T2-weighted MRI showing large effusions and numerous lesions in the spine and the spleen. (**C**) Maximum-intensity projection of coronal T1-weighted gradient echo sequence after injection of contrast into liver lymphatics showing leak of contrast into the duodenum and abnormal lymphatic drainage into the thorax (short arrows)

GLA is a congenital multifocal lymphatic dysplasia with proliferation of dilated and/or cystic lymphatic channels involving mediastinal or retroperitoneal soft tissues, bones, and abdominal viscera, particularly the spleen. Imaging features are best depicted on fat-suppressed fluid-sensitive MRI sequences. GLA may be associated with variants in PIK3CA and usually lacks the aggressive clinical course and coagulopathy characteristic of KLA. KLA may share the imaging features of GLA (Fig. 6) but is more aggressive and is often associated with hemorrhagic effusions and coagulopathy; variants in NRAS have been described. GSD is characterized by aggressive soft-tissue and osseous lymphatic malformation with progressive segmental osteolysis, resulting in so-called vanishing bone disease. Any of these entities may present with lymphatic leakage (Fig. 7). CCLA comprises functional and anatomical disorders of the central lymphatic channels, including the TD, that cause chylous effusions, PB, or PLE without extensive infiltrative malformations [38–40].

**Fig. 8:**
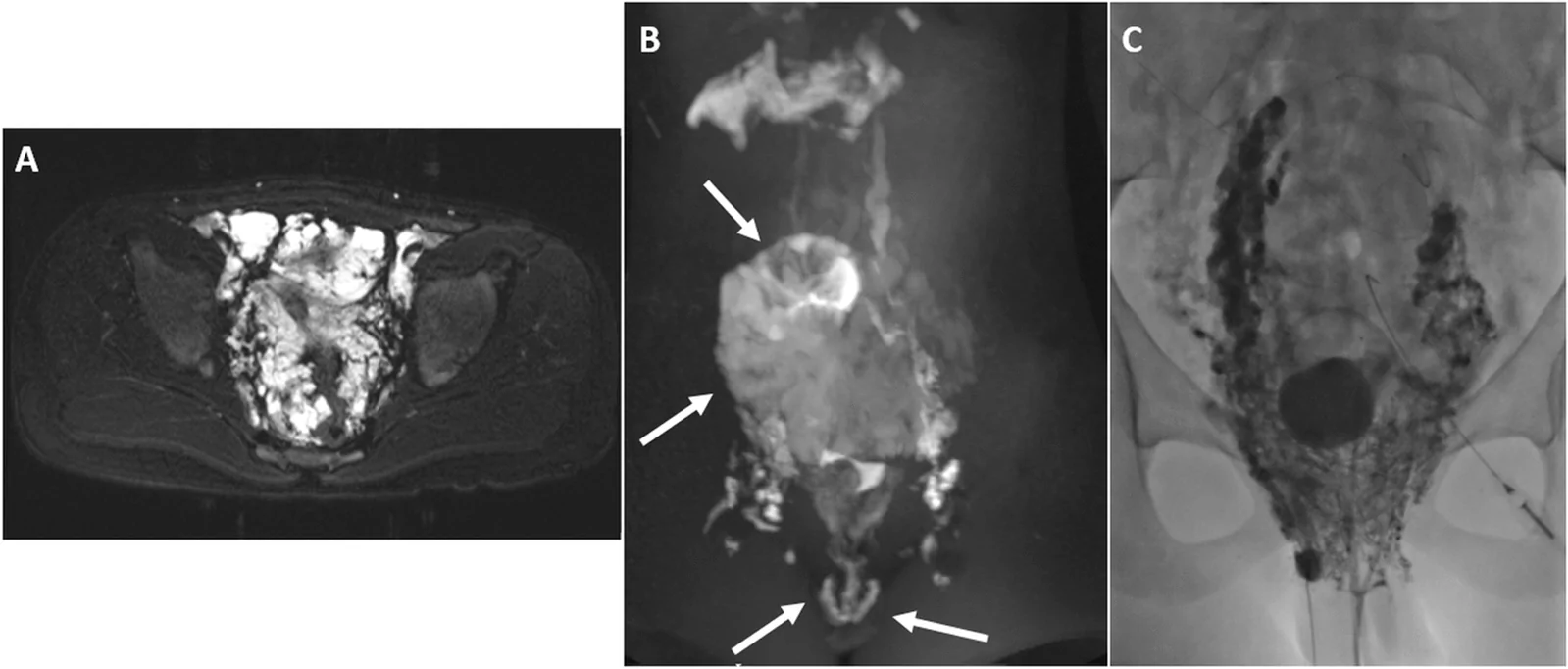
12year-old female with generalized lymphatic anomaly and vaginal leaking. (**A**) Axial T2-weighted MRI showing diffuse pelvic lymphatic malformation. (**B**) Maximum-intensity projection of coronal T1-weighted gradient echo sequence after injection of contrast into mesenteric and inguinal lymphatics showing perfusion of the labia (arrows) and filling of lymphatic cysts in the abdomen and pelvis. Note the dilated thoracic duct. (**C**) Fluoroscopic imaging demonstrating filling of abnormal channels with sclerosant and glue. Patient’s symptoms had resolved by the time of publication

In patients with complex lymphatic anomalies, DCMRL provides detailed assessment of anatomy and dysfunction and is a prerequisite for planning an intervention. When interventional treatment is indicated, selective embolization of abnormal or leaking lymphatic channels is the preferred approach. Target selection can be performed with a microcatheter after antegrade or retrograde TD access, and embolization may be performed with nBCA or a sclerosant (Fig. 8) [2, 41].

As with venous and arteriovenous malformations, the guiding principle is penetration of the target network or nidus with the embolic agent. Coils may be placed in the selected lymphatic network as a backstop. In complex lymphatic anomalies, complete TD embolization occludes the principal lymphatic outlet and may redirect lymph into other body compartments, thereby worsening the disease. It also limits future access options, analogous to proximal embolization of an arteriovenous malformation. Complete TD embolization should therefore be avoided unless no selective or decompressive alternative is available [30, 42].

**Fig. 9:**
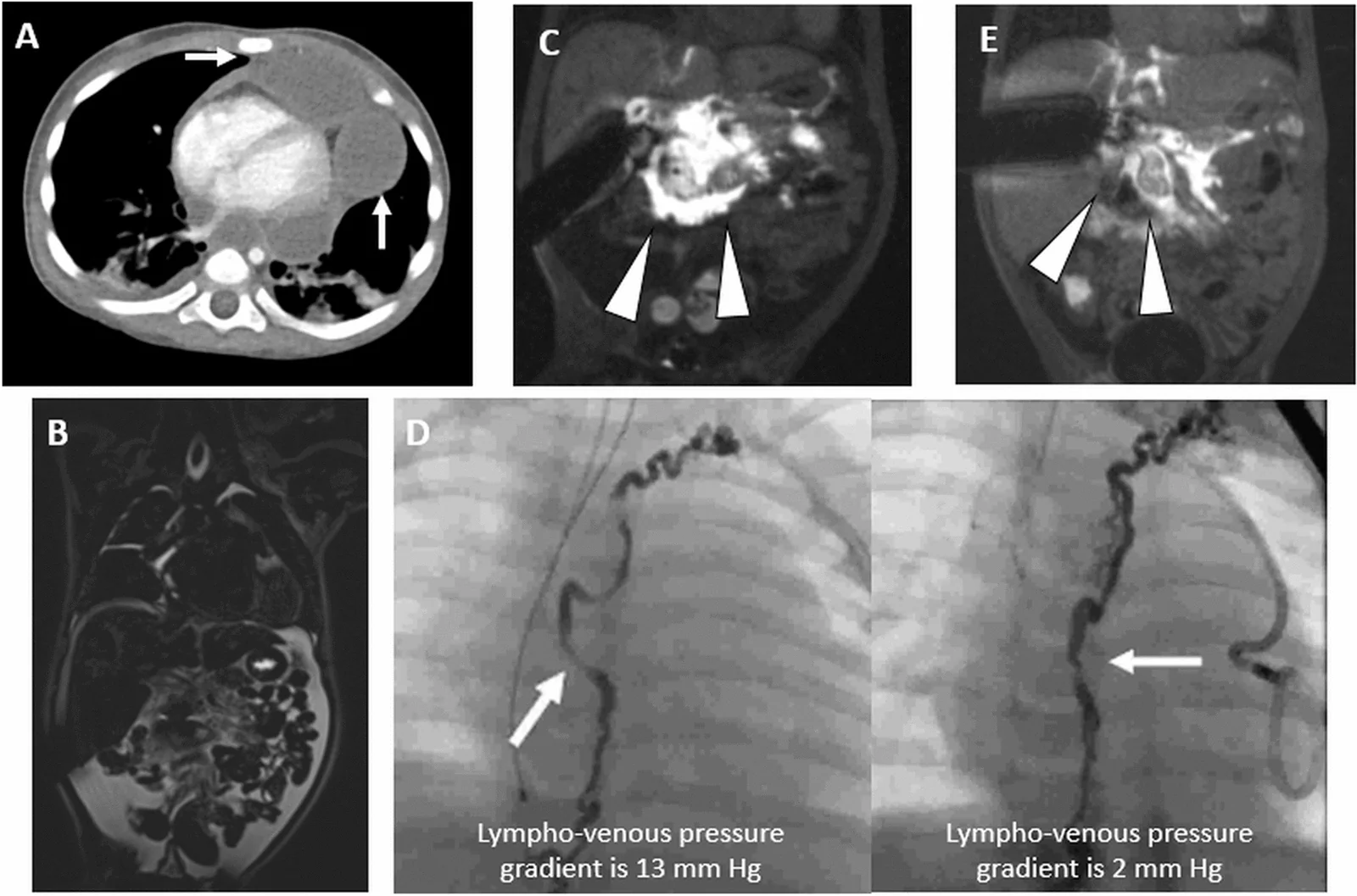
10-month-old female with generalized lymphatic anomaly, chylous ascites, protein-losing enteropathy, and lymphatic malformations of the kidney and mediastinum. (**A**) Axial CT image showing lymphatic malformation of the mediastinum/pericardium (short arrows). (**B**) Coronal T2-weighted MRI showing ascites. (**C**) Coronal T1-weighted gradient echo image after injection of contrast into liver lymphatics showing leak of contrast into duodenum (arrowheads). (**D**) There is mass effect and obstruction on the thoracic duct that resolves after drainage of the large lymphatic malformation (arrows) with corresponding decrease in the lympho-venous pressure gradient. (**E**) Follow-up coronal T1-weighted gradient echo image after injection of contrast into liver lymphatics showing leak of contrast into duodenum has resolved (arrowheads)

**Fig. 10:**
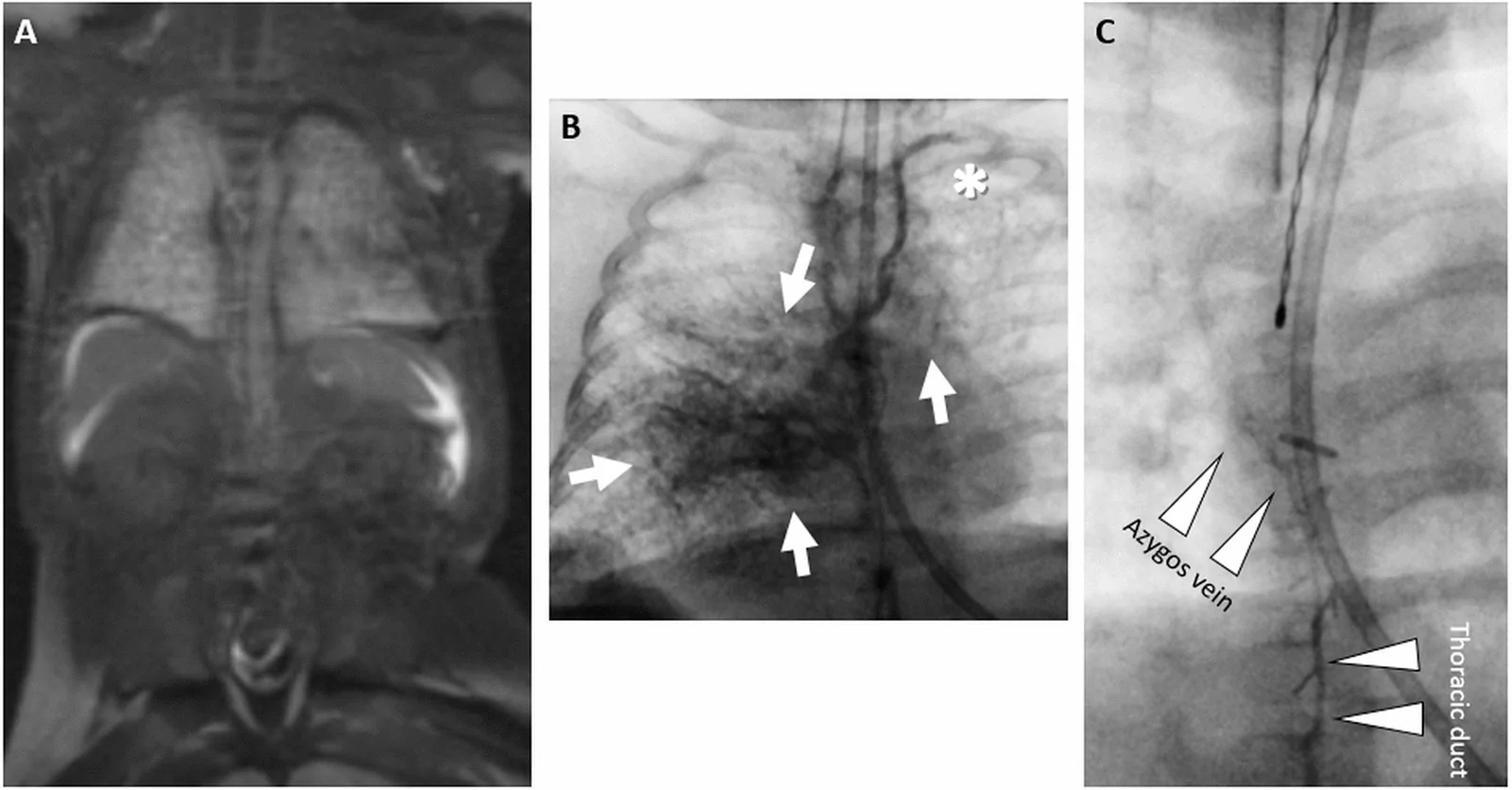
3-month-old boy after repair of supracardiac total anomalous pulmonary venous connection. (**A**) Coronal T2-weighted MR image showing dense pulmonary edema from lymphatic perfusion, as well as ascites. (**B**) Fluoroscopic lymphangiogram showing pulmonary lymphatic perfusion (arrows) with no outflow into the central veins (asterisk). (**C**) Lymphangiogram after end-to-side anastomosis of the thoracic duct to the azygous vein showing contrast flow from the duct into the vein (arrowheads)

In patients with a suspected intrinsic TD stenosis, balloon angioplasty may be considered when a lymphovenous pressure gradient has been documented [43, 44]. Empirical angioplasty of the TD outlet risks injury and worsening of symptoms. Extrinsic obstruction may be caused by crossing vessels or masses (Fig. 9). If TD obstruction cannot be treated percutaneously, surgical lymphovenous anastomosis may be considered (Fig. 10). TD stenting for lymphatic outflow obstruction has also been described [45], but long-term patency, particularly in children, remains uncertain because durable follow-up data are not yet available.

**Fig. 11:**
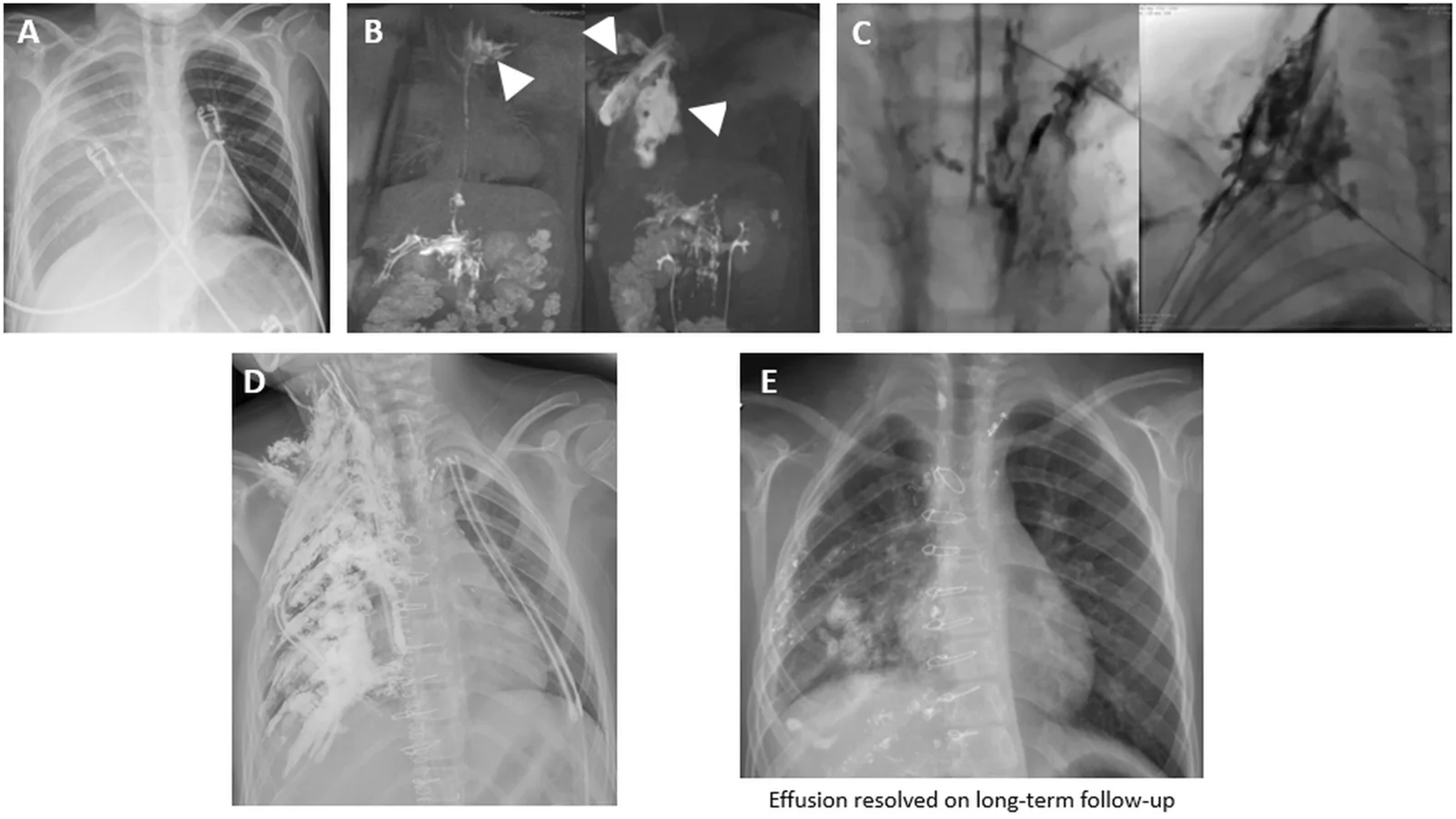
11-year-old male with generalized lymphatic anomaly and severe chylothorax-chylopericardium. (**A**) Chest radiograph showing a large right pleural effusion. (**B**) Coronal T1-weighted gradient echo MRI after injection of contrast into inguinal lymphatics and right supraclavicular networks showing abnormal perfusion and leak into the thorax (arrows). (**C**) These and many other networks were embolized over multiple sessions, but the effusions persisted. (**D**) The patient then underwent a combined procedure with open surgical pleurodesis, excision of lymphatic malformation and glue embolization of deep networks. (**E**) He is symptom free on long-term follow-up

The burden of complex lymphatic anomalies may be too extensive for percutaneous treatment alone. In these settings, a combined interventional and surgical approach may be required (Fig. 11). When cystic components contribute to symptoms, percutaneous sclerotherapy with doxycycline, bleomycin, and/or ethanol may provide sustained benefit [46, 47].

A multidisciplinary approach integrating diagnostic imaging, interventional radiology, supportive medical management, surgery, and gene profiling is essential for these severe disorders [40]. Interventional strategies may need to be complemented by targeted medical therapy guided by an identified pathogenic variant. Genetic analysis may be performed on tissue biopsy material when accessible, fluid obtained during drainage, or plasma cell-free DNA. Drug classes currently used include mTOR inhibitors, direct PIK3CA inhibitors, and MEK inhibitors [48–50]. These agents have shown efficacy in remodeling the central lymphatic system and reducing lymphatic proliferation and leakage (Fig. 6) [51]. Ongoing research may enable more individualized treatment and clarify the genetic basis of these diseases.

#### Lymphatic Flow Disorders in Chronic Lung Disease

**Fig. 12:**
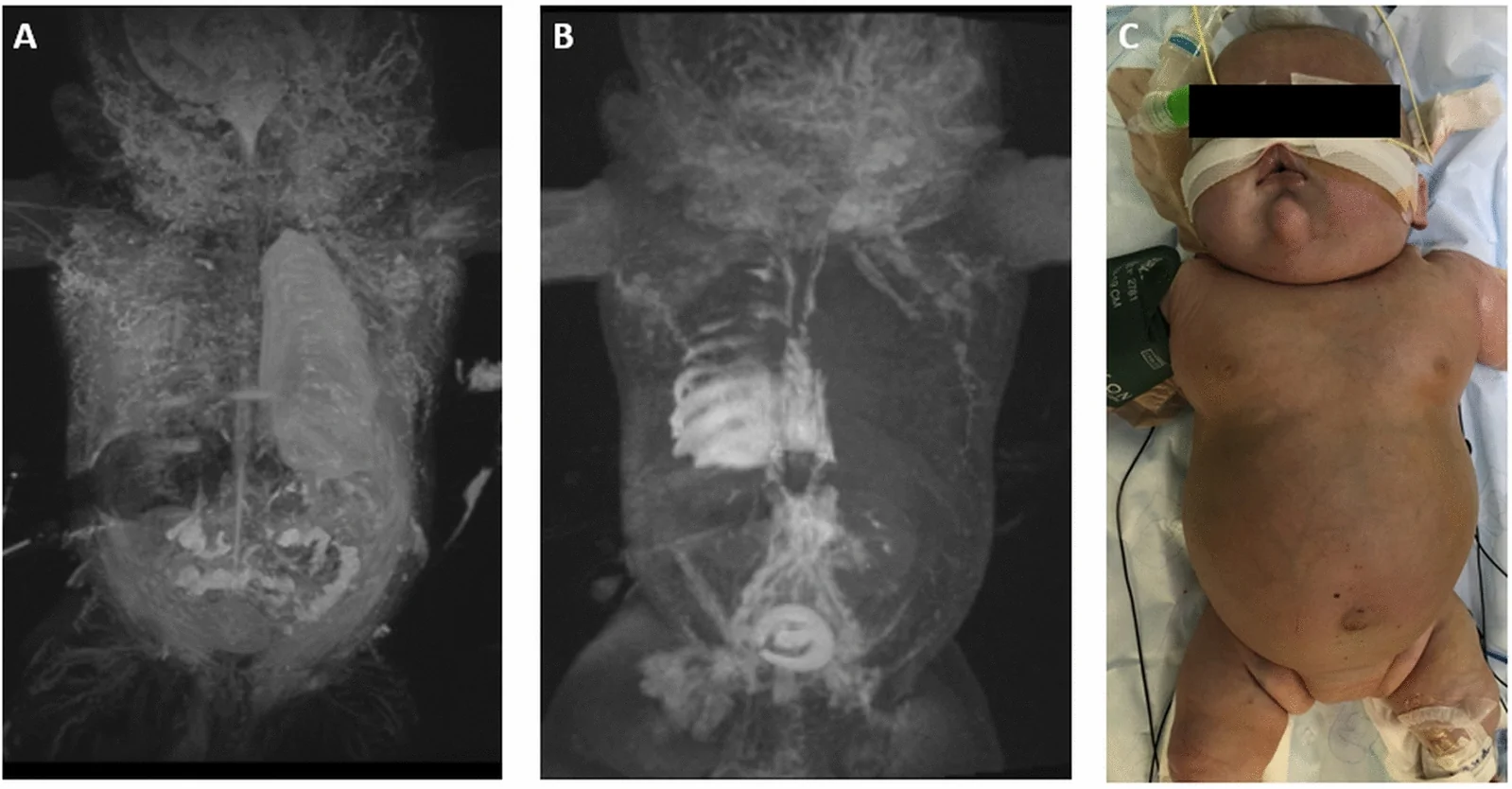
5-month-old male with bronchopulmonary dysplasia. (**A**) Maximum-intensity projection of coronal T2-weighted MRI showing severe proliferation of subcutaneous lymphatic networks in the chest and head, large effusions, and ascites. (**B**) Maximum-intensity projection of coronal T1-weighted gradient echo sequence after injection of contrast into inguinal lymphatics showing leak of contrast into the right pleural space and dermal perfusion, especially of the head. (**C**) Photograph of a similar child showing anasarca

Chronic lung disease of prematurity, or bronchopulmonary dysplasia (BPD), is increasingly recognized as not only a pulmonary condition, but also one with systemic effects, including on the lymphatic system. Lymphatic disorders in BPD often manifest due to elevated intra-thoracic impairing lymphatic drainage due to increased lymphatic afterload. Infants with severe BPD frequently require prolonged positive pressure ventilation due to poor lung mechanics, and this raises intrathoracic pressure and thus central venous pressure, thereby impeding TD outflow. This can result in lymphatic congestion and further decrease in lung compliance, creating a positive feedback loop that exacerbates lymphatic failure. In these cases, common manifestations are severe anasarca (often involving the head) and pleural effusions, with ascites and PLE developing as the disease progresses (Fig. 12).

Lymphangiographic assessment of the TD has revealed that high levels of positive end-expiratory pressure (PEEP) can severely reduce or halt TD outflow in these patients. As reducing PEEP restores lymphatic drainage, pressure-induced obstruction at the TD outlet is thought to compromise fluid clearance. Furthermore, some patients with BPD may have underlying lymphatic dysplasia, compounding the effects of ventilator-induced pressures. Optimizing ventilator settings to minimize PEEP while preserving gas exchange may improve outcomes [52, 53]. Interventional procedures, including selective embolization and lymphatic externalization, have demonstrated clinical benefit in seleced cases, but successful management of the underlying disease is necessary for optimal control of this severe lymphatic disorder.

## Considerations in Pediatric Patients

Imaging and treatment of lymphatic flow disorders in neonates and children require pediatric-specific adaptations. Equipment size, radiation exposure, contrast dose, anesthesia or sedation, and transport between procedural and imaging suites must be adjusted to age, body size, and clinical status. Breath-holding is often not feasible in infants and young children, favoring respiratory-triggered or free-breathing MR techniques; CT and fluoroscopic protocols must be strictly dose optimized. Particular attention must be paid to cardiovascular physiology and right-to-left shunts in neonatal and CHD populations. Preprocedural review of the cardiac and venous anatomy is essential to avoid serious complications, including stroke [27]. Postprocedural surveillance should address hemorrhage, sepsis, thrombosis, and deterioration of lymphatic outflow.

## Conclusion

In children, the central lymphatic system may be affected by a broad spectrum of primary and secondary lymphatic disorders associated with severe clinical presentations. With a growing diagnostic and therapeutic armamentarium, it is essential for radiologists to be familiar with these disorders, imaging modalities (notably DCMRL with multi-compartment assessment), interventional treatment options, and the need for multidisciplinary management. All these are beneficial to inform a vigilantly planned intervention. As the underlying pathophysiology can be complex, non-selective embolizaton of central lymphatics can lead to considerable worsening of the disease.
